# The Effect of Different Mixing Methods on Working Time, Setting Time, Dimensional Changes and Film Thickness of Mineral Trioxide Aggregate and Calcium-Enriched Mixture

**DOI:** 10.7508/iej.2015.04.008

**Published:** 2015

**Authors:** Shahriar Shahi, Negin Ghasemi, Saeed Rahimi, Hamidreza Yavari, Maryam Janani, Hadi Mokhtari, Mahmood Bahari, Parastu Rabbani

**Affiliations:** a *Dental and Periodontal Research Center, Dental School, Tabriz University of Medical Sciences, Tabriz, Iran; *; b * Department of Endodontics, Dental School, Tabriz University of Medical Sciences, Tabriz, Iran; *; c * Department of Restorative Dentistry, Dental School, Tabriz University of Medical Sciences, Tabriz, Iran, *; d *Private Practice, Tabriz, Iran*

**Keywords:** Calcium-Enriched Mixture, Dimensional Changes, Film Thickness, Mineral Trioxide Aggregate, Setting Time, Working Time

## Abstract

**Introduction:**

The aim of the present study was to evaluate the effect of different mixing techniques (conventional, amalgamator and ultrasonic mixing) on the physical properties the working time (WT), setting time (ST), dimensional changes (DC) and film thickness (FT)] of calcium-enriched mixture (CEM) cement and mineral trioxide aggregate (MTA).

**Methods and Materials:**

The mentioned physical properties were determined using the ISO 6786:2001 specification. Six samples of each material were prepared for three mixing techniques (totally 36 samples). Data were analyzed using descriptive statistics, two-way ANOVA and Post Hoc Tukey’s tests. The level of significance was defined at 0.05.

**Results:**

Irrespective of mixing technique, there was no significant difference between the WT and FT of the tested materials. Except for the DC of MTA and the FT of the all materials, other properties were significantly affected with mixing techniques (*P*<0.05). The ultrasonic technique decreased the ST of MTA and CEM cement and increased the WT of CEM cement (*P*<0.05).

**Conclusion:**

The mixing technique of the materials had no significant effect on the dimensional changes of MTA and the film thickness of both materials.

## Introduction

Ideal physical properties of an endodontic biomaterial are essential prerequisites for their successful clinical application. To achieve these ideal properties in hydraulic cements, the particles should be completely mixed with water. In fact, the technique used to mix these materials provides a proper contact between the powder particles and the liquid [1-3]. 

Mineral trioxide aggregate (MTA) and calcium-enriched mixture (CEM) cement are biomaterials with different calcium compounds. CEM cement consists of calcium oxide, calcium phosphate, calcium carbonate, calcium silicate, calcium sulfate, calcium hydroxide and calcium chloride [4-6]. Based on the results of previous studies, the mean setting time (ST) of MTA is 165 min, which is longer than that of its major constitute (Portland cement) due to lower contents of sulfur and tricalcium aluminate [5, 7]. The setting time of CEM cement is shorter and is almost 1 h [5]. The working time (WT) of these materials is almost 5 min [8]. CEM cement has lower film thickness compared to MTA [4]. However, similar dimensional changes (DC) have been reported in CEM cement and MTA, with setting expansion [4]. 

These cements are hydrophilic; therefore the powder-to-liquid ratio affects the properties of the resultant mix. On the other hand, the mixing technique affects the hydration degree [2, 9] .The mechanical mixing technique with the use of an amalgamator can decrease the number of air bubbles between the adjacent powder particles, which results in complete wetting of the particles, thus increasing the homogeneity of the mixture [1]. Based on the results of previous studies, use of an amalgamator improves the compressive strength of the mixed cement [1]. 

Use of ultrasonic energy has attracted attention for mixing materials in many studies because it increases interaction of particles through extending the surface area of the particles that take part in setting [1, 2]; as a result it decreases the ST and increases the compressive strength, surface microhardness and the density of the resultant mixture [1-3, 10]. 

Shahi *et al.* [3] reported that the push-out bond strength of MTA was not affected by different mixing techniques. In another study, Shahi *et al.* [10] evaluated the effects of different mixing techniques on the compressive strength and flow rates of MTA and CEM cement and reported the highest compressive strength with the use of the ultrasonic technique. However, MTA and CEM cement exhibited the highest flow rates with the use of amalgamator and hand mixing techniques, respectively. 

The aim of the present study was to evaluate the effect of different mixing techniques, *i.e.* conventional (hand), amalgamator and ultrasonic mixing, on WT, ST, DC and film thickness (FT) of CEM cement and MTA.

## Materials and Methods

***Preparation of samples***

Before mixing procedures, the mixing pads, spatulas and the glass slabs were kept at 23±1^°^C temperature for 1 h. The powders and liquids were mixed using conventional, amalgamator and ultrasonic techniques. In the conventional technique, the powder and the liquid were mixed by hand according to manufacturer’s instructions. In the ultrasonic technique, an ultrasonic scaling device (Juya Electronics, Iran) was used for mixing. In the amalgamator technique, proper proportions of the powder and liquid were placed in the amalgamator container (Duomat II, Dental und Goldhalbzeug, 600 Frankfurt, Germany) and mixed for 20 sec.

***Setting time***

The ST of CEM cement (Yektazist Dandan, Tehran, Iran) and MTA (Angelus, Londrina, Paraná, Brazil) was determined as recommended by ISO 6786:2001 specification [2, 10]. The materials were mixed and placed in cylindrical metallic molds measuring 15 mm in diameter and 5 mm in height (a total of 36 molds) including 6 molds for each material and for each mixing method. Within 2 min after initiation of mixing, the cylinders were placed in an incubator under a relative humidity of 95‒100% at 37^°^C. After 30 sec to 1 min, the special plunger of the Vicat penetrometer (Schiller Park, Illinois, USA) was inserted on the surface of each material in a perpendicular manner with a crosshead speed of 1 mm/min and maintained for 5 sec. The procedure was repeated every 30 sec until the plunger was unable to create a completely circular indentation on the surface of the samples. ST was defined as the time elapsed since the beginning of mixing until the plunger was unable to create a completely circular indentation on the surface of the material.

***Working time***

Based on ISO 6876:2001, recommended proportions of the powder and liquid of each material were mixed using the different mixing techniques. Then 0.5 mL of the mixtures was placed in the center of a glass slab and then another slab was placed on it, with a 100-g weight placed on it for 10 min. There were 6 samples for each material for three mixing methods. Then the diameter of the sample was measured and the time necessary for its diameter to reach a value 10% less than the original diameter was recorded as the WT.

***Dimensional changes***

ISO 6876:2001 was used to determine the physical properties [10]. Correct proportions of the materials were mixed using the three techniques mentioned above to prepare 6 samples for test materials for three mixing methods, measuring 12 mm in height and 6 mm in diameter, within Teflon molds. Each mold was placed on a glass slab, measuring 75×25×1 mm, which was covered with a cellophane tape. The molds were a little overfilled and then a microscope plate covered with cellophane tape was placed on the top of each sample. This set was gently held by a C-shaped pincer. Five min after initiation of mixing, the set was transferred into an incubator under a relative humidity of 95% at 37^°^C for a duration, 3 times longer than that recommended by the manufacturer for setting. After retrieval of samples from the incubator, both ends of the mold were abraded with a wet piece of 600-grit silicon carbide paper to produce a flat and homogeneous surface. The samples were removed from the molds and a digital Vernier measuring tool, accurate to 0.01 mm, was used to determine their lengths. In the next stage, the samples were placed in a glass vial containing 30 mL of deionized distilled water for 30 sec under 95% relative humidity for 30 days. Then the samples were dried with absorbent paper and their lengths were measured again. The percentages of DC were calculated using the following formula: (L30-L)/L×100 in which L30 is the length of the sample after 30 days and L is the initial length of the sample. Then the mean value of 5 samples for each mixing technique was considered as the mean DC of the sample.

***Film thickness***

The film thickness of the materials was measured based on ISO 6876:2001 specifications. The overall thickness and surface area of the two glass slabs (5 mm and 200 mm^2^, respectively) was measured with a micrometer accurate to 1 µm, under a relative humidity of 95% at 37^°^C, the materials were placed between the two glass slabs after mixing with the technique described above. Three min after initiation of mixing, the glass slabs were placed in the IMI device (IMI Norgen Inc., Littleton, CO, USA) under a 150-N force so that the material was completely distributed between the two slabs. After 10 min, the thickness of the whole set was measured using a micrometer. The test was repeated 3 times for each material and each mixing technique. 

***Statistical Analysis***

Data were analyzed using descriptive statistics (mean±SD). Shapiro-Wilk test was used to evaluate normal distribution of data. Since the data was normally distributed, the two-way ANOVA was used to evaluate the significant effect of the material type and mixing methods. The post hoc Tukey’s test was used for the two-by-two comparison of the groups. SPSS software (SPSS version 18.0, SPSS, Chicago, IL, USA) was used for the analysis of data. The level of statistical significance was defined at 0.05.

## Results

Evaluation of the physical properties of the materials revealed the following results (Table 1): irrespective of the mixing technique, there were no significant differences in the means of WT and FT of CEM cement and MTA (*P*<0.05). The DC of MTA, was significantly higher than CEM cement. The setting time of CEM cement was significantly shorter than MTA (*P*<0.05). The mixing technique of the materials had no significant effect on the DC of MTA and the FT of both materials (*P*>0.05). With the use of the ultrasonic mixing technique, the WT of CEM cement significantly decreased (*P*<0.05). The ST of CEM cement and MTA decreased significantly with the use of amalgamator and ultrasonic mixing techniques compared to the hand mixing method (*P*<0.05). The DC of CEM cement with the use of amalgamator and hand mixing techniques were in the form of shrinkage, with no significant differences; however, the ultrasonic mixing technique resulted in the expansion of this material (*P*>0.05). 

## Discussion

The aim of the present study was to evaluate the effect of different mixing techniques of CEM cement and MTA on some selective physical properties. The results showed that the ST of MTA and CEM cement and the WT of CEM cement was significantly affected by the mixing technique. 

The mixing technique of hydrophilic cements is one of the factors affecting the physical properties of these materials [9]. The ST is considered as one of the most important physical properties of each material and is defined as the duration of time a material needs to become rigid from a waxy or a liquid consistency [11].

Long ST has always been considered as one of the disadvantages of MTA. On the other hand, after apical surgeries the retro-filling materials might be contaminated and washed away in contact with the influence of tissue fluids [12]. Although the ST of CEM cement is shorter than MTA, it is still impossible to carry out one-visit treatment procedures [4, 8]. Clinically, a ST of 25‒30 min is considered favorable [13]. In fact, when a material sets fast there is a short time for its contamination in the oral cavity; on the other hand, the increase in initial strength , decreases the odds of its being washed-out. As a result, the restorative material can safely be placed over it in the same session [13, 14]. More specifically, any change in the setting process of bioactive materials, including the time and production of reaction products, which are mainly calcium and hydroxyl ions, might affect the production of hydroxyapatite layer and the bioactivity of these materials [7]. The mixing technique, the amount of liquid used, the force used for packing and the environmental moisture affect the setting process [14-16]. In the present study, all the variables were matched in all the samples except for the mixing technique. In this context, the powder-to-liquid ratio was 3:1 in all the samples; placement and packing of the materials were carried out by one operator and all the samples were stored under the same environmental conditions and moisture until used for the purpose of the study. 

In the present study, the ST of MTA and CEM cement decreased to clinically acceptable levels with the application of amalgamator and ultrasonic mixing techniques. The decrease in ST with the ultrasonic technique is consistent with a previous study. On the other hand, the overall setting time of CEM cement was less than that of MTA, irrespective of the mixing technique, consistent with the results of the studies by Asgary *et al.* [4]. Given the smaller powder particles of CEM cement compared to MTA, which increases the hydration rate, the shorter ST is expected. 

DC is influenced by ST and an increase in ST results in a decrease in dimensional stability [16] . Slow expansion during the setting reaction can increase the adaptation of the material with the cavity walls in the area, improving the seal; however, rapid expansion of the root-end filling material might result in the formation of cracks in the thin walls at the apical end. On the other hand, shrinkage results in the loss of marginal adaptation, leading to leakage [4, 11, 13, 17]. 

**Table 1:** Mean (SD) of studied variable in the groups considering the mixing techniques (^a^. shrinkage)

**Physical properties**	**Material**	**Conventional **	**Ultrasonic **	**Amalgamator**
**Film thickness (mm) **	CEM	1.3 (0.05)	1 (0.02)	0.8 (0.03)
	MTA	1.3 (0.05)	1.1 (0.03)	1.1 (0.01)
**Dimensional changes (mm)**	CEM	-0.34 (0.03)	0.1 (0.13)	-0.28 (0.05 ^a^)
	MTA	0.3 (0.09)	0.4 (0.09)	0.35 (0.01)
**Working time (h)**	CEM	5:38:58 (0:2:8)	12:8:53 (0:37:3)	5:56:34 (0:17:13)
	MTA	6:8:23 (0:2:54)	5:36:54 (2:41:54)	5:58:48 (0:19:5)
**Setting time (h)**	CEM	0:52:50 (0:0:36)	0:36:45 (0:0:31)	0:0:30 (0:0:50)
	MTA	0:35:40 (0:0:36)	0:27:45 (0:0:54)	0:23:40 (0:0:28)

The DC depends on the soaking properties of the material [7, 13] and since MTA is more porous than CEM cement, it absorbs more fluids and its DC are in the form of expansion other than shrinkage compared to MTA which is consistent with the results of the present study and it seems that an expansion of the elements such as gypsum is responsible for the higher DC of MTA.

In these two materials, the DC was in the form of shrinkage except for the CEM/ultrasonic group. Therefore, by considering the DC factor in a retrofilling material, it appears the CEM/ultrasonic and MTA/all mixing groups can be considered suitable for use in root-end surgeries. On the other hand, the mixing technique of CEM cement should be taken into account when it is used as a retro-filling material. 

Another variable evaluated in the present study was the WT which is defined as the time during which the material can be manipulated clinically [8]. In relation to the materials used in the present study the numeric value for this variable in previous studies was around 5 min, which was almost the same in the present study. In general, the mean WT was almost the same for all the three materials, irrespective of the mixing technique. An important consideration was the two-fold increase in the WT of CEM cement with ultrasonic mixing technique. 

The FT was another variable evaluated in the present study, which was higher for MTA compared to CEM cement, consistent with the results of a study by Asgary *et al. *[4]. This variable was the only variable that was not influenced by the mixing technique.

## Conclusion

In summary, by considering the values obtained for the physical properties of the evaluated materials and the effect of different mixing techniques on these properties, the positive effect of the ultrasonic mixing technique on some important properties, such as increased working time and a decreased setting time should not be ignored. On the other hand, the dimensional changes of CEM cement with this mixing technique shifted from shrinkage to expansion. 
